# Beyond CSF and Neuroimaging Assessment: Evaluating Plasma miR-145-5p as a Potential Biomarker for Mild Cognitive Impairment and Alzheimer’s Disease

**DOI:** 10.1021/acschemneuro.3c00740

**Published:** 2024-02-26

**Authors:** Qingfeng Wen, Mandy Melissa Jane Wittens, Sebastiaan Engelborghs, Marcel H. M. van Herwijnen, Maria Tsamou, Erwin Roggen, Bert Smeets, Julian Krauskopf, Jacco Jan Briedé

**Affiliations:** † Department of Toxicogenomics, Maastricht University, Universiteitssingel 50, 6229 ER Maastricht, The Netherlands; ‡ MHeNS, School for Mental Health and Neuroscience, Maastricht University, Universiteitssingel 50, 6229 ER Maastricht, The Netherlands; § Department of Biomedical Sciences, Institute Born-Bunge, University of Antwerp, Universiteitsplein 1, BE-2610 Antwerpen, Belgium; ∥ Neuroprotection and Neuromodulation (NEUR), Center for Neurosciences (C4N), Vrije Universiteit Brussel (VUB), Laarbeeklaan 103, 1090 Brussel, Belgium; ⊥ Department of Neurology, Universitair Ziekenhuis Brussel (UZ Brussel), Laarbeeklaan 101, 1090 Brussel, Belgium; # ToxGenSolutions (TGS), 6229EV Maastricht, The Netherlands

**Keywords:** Alzheimer’s disease, biomarkers, microRNAs, miR-145, PI3K/AKT signaling

## Abstract

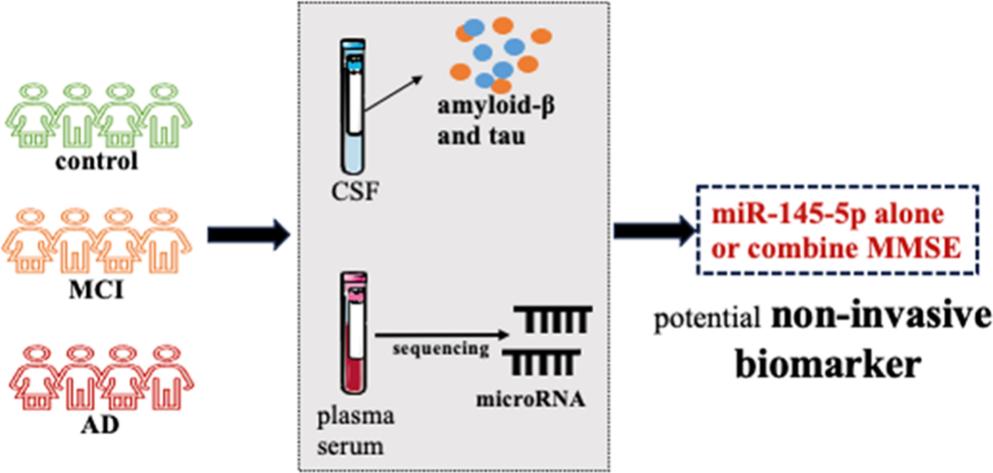

Alzheimer’s disease (AD) is the most common cause of dementia. New strategies for the early detection of MCI and sporadic AD are crucial for developing effective treatment options. Current techniques used for diagnosis of AD are invasive and/or expensive, so they are not suitable for population screening. Cerebrospinal fluid (CSF) biomarkers such as amyloid β1–42 (Aβ1–42), total tau (T-tau), and phosphorylated tau181 (P-tau181) levels are core biomarkers for early diagnosis of AD. Several studies have proposed the use of blood-circulating microRNAs (miRNAs) as potential novel early biomarkers for AD. We therefore applied a novel approach to identify blood-circulating miRNAs associated with CSF biomarkers and explored the potential of these miRNAs as biomarkers of AD. In total, 112 subjects consisting of 28 dementia due to AD cases, 63 MCI due to AD cases, and 21 cognitively healthy controls were included. We identified seven Aβ1–42-associated plasma miRNAs, six P-tau181-associated plasma miRNAs, and nine Aβ1–42-associated serum miRNAs. These miRNAs were involved in AD-relevant biological processes, such as PI3K/AKT signaling. Based on this signaling pathway, we constructed an miRNA-gene target network, wherein miR-145-5p has been identified as a hub. Furthermore, we showed that miR-145-5p performs best in the prediction of both AD and MCI. Moreover, miR-145-5p also improved the prediction performance of the mini-mental state examination (MMSE) score. The performance of this miRNA was validated using different datasets including an RT-qPCR dataset from plasma samples of 23 MCI cases and 30 age-matched controls. These findings indicate that blood-circulating miRNAs that are associated with CSF biomarkers levels and specifically plasma miR-145-5p alone or combined with the MMSE score can potentially be used as noninvasive biomarkers for AD or MCI screening in the general population, although studies in other AD cohorts are necessary for further validation.

## Introduction

Alzheimer’s disease (AD) is the most common neurodegenerative disease and a leading cause of death in older people.^1^ Early identification of sporadic AD is critical for prevention and the development of effective therapies. According to the National Institute on Aging-Alzheimer’s Association (NIA-AA) criteria, there are three disease stages of AD including preclinical stage, mild cognitive impairment (MCI) due to AD, and dementia due to AD. At the preclinical stage, there are opportunities to diagnose AD since measurable biomarker changes in the brain may occur years before symptoms are detected.^2^ In MCI, mild changes in memory and thinking are noticeable and can be measured by means of a neuropsychological examination, but subjects still maintain the ability to independently perform their daily life. Besides, subjects living with MCI who have the hallmark changes for AD in the brain are considered an early symptomatic stage of the disease continuum for AD, which is generally indicated as MCI due to AD.^3^

Neurodegeneration in the brain in AD can be detected using computed tomography (CT), positron emission tomography (PET), and magnetic resonance imaging (MRI) brain scans. MRI features, including hippocampal volumetry, have been explored as potential biomarkers for AD diagnosis.^4^ Classical neurobiological hallmarks of AD are amyloid plaques and neurofibrillary tangles. The main component of amyloid plaques includes amyloid-β1–42 (Aβ1–42), and the principal component of neurofibrillary tangles includes phosphorylated tau181 (P-tau181), the levels of which can be detected in cerebrospinal fluid (CSF) biomarkers following lumbar puncture. The CSF biomarkers Aβ1–42, total tau (T-tau), and P-tau181 levels are core biomarkers in the early diagnosis of AD.^5−10^ These become abnormal ones or maximal two decades before symptom onset.^11,12^ Furthermore, changes in cognitive function resulting in cognitive impairment can be screened via neuropsychological examination such as Alzheimer’s disease assessment scale-cognitive (ADAS-Cog) and the mini-mental state examination (MMSE). However, except for screening via neuropsychological examination, current alternatives are expensive, invasive, and/or only available in highly specialized centers and, thus, not suitable for the screening in large populations.

Therefore, it is essential to identify cheap, easily detectable, and sensitive biomarkers specifically for the AD risk that can be obtained in a minimally invasive manner. Circulating biomarkers that can easily, rapidly, and cost effectively be detected in available biofluids other than CSF, such as blood, urine, or tear fluid are being developed and have become promising in fulfilling this role.^13^ However, no such biomarker is currently available in clinical practice. In fact, although there is an association between amyloidosis and cognitive decline, whether Aβ1–42 levels in plasma samples correlate well with AD is still debated.^13,14^ Furthermore, quantifying plasma Aβ1–42 or T- or P-tau181 has become analytically challenging due to small dynamic ranges.^15,16^ Considering the limitations of the amyloid-β cascade hypothesis and tau protein hypothesis, novel candidate biomarkers have emerged, such as oxidative stress biomarkers.^17,18^ It is well known that the brain in AD is under increased oxidative stress, leading to damage in membranes, proteins, and nucleic acids.^19,20^ However, as oxidative stress is a commonality in the pathophysiology of neurodegenerative diseases, oxidative stress biomarkers may lack specificity for AD.^21,22^

Growing evidence suggests that microRNAs (miRNAs), which are a class of noncoding RNAs that regulates gene expression at the post-transcriptional level, can be potential biomarkers for AD.^23−27^ Moreover, it has been shown that excreted circulating miRNAs can cross the blood–brain barrier,^28^ indicating its potential as biomarkers for neurodegenerative diseases. Additionally, miRNAs have been implicated in neuronal apoptosis regulation and attenuation of Aβ neurotoxicity^29,30^ and it has been reported that plasma miRNAs are altered prior to the occurrence of symptoms.^31^ Several studies have focused on identifying consistent circulating miRNA signature for AD;^32−35^ however, so far, few results were concordant.

Considering the important role of CSF biomarkers in early detection of AD, we aimed to identify blood-circulating miRNAs associated with the levels of the CSF biomarkers Aβ1–42, T-tau, and P-tau181. To achieve this, we employed a linear mixed model that considered age, gender, education, and medication—factors that might affect cognitive function in AD.^36−38^ We included plasma- and serum-circulating miRNAs by an unbiased approach of next-generation sequencing from 112 participants including AD, MCI due to AD, and not age-matched but cognitively healthy control subjects. Subsequently, we explored the potential of these CSF biomarker-associated miRNAs as biomarkers of AD and MCI through receiver operating characteristic (ROC) analysis. Moreover, the combination of our miRNA biomarker and the cognitive impairment measurement obtained via the MMSE score was investigated for its predictive value. We validated the expression of these miRNAs and their gene targets in brain tissue-derived miRNAs and mRNA (mRNA) expression data from five AD patients and two control subjects. The potential biological processes in which these miRNAs are involved were explored. Additionally, plasma miRNA RT-qPCR data from GSE90828^39^ were used as an external validation dataset in our analysis.

## Results

**Figure 1:**
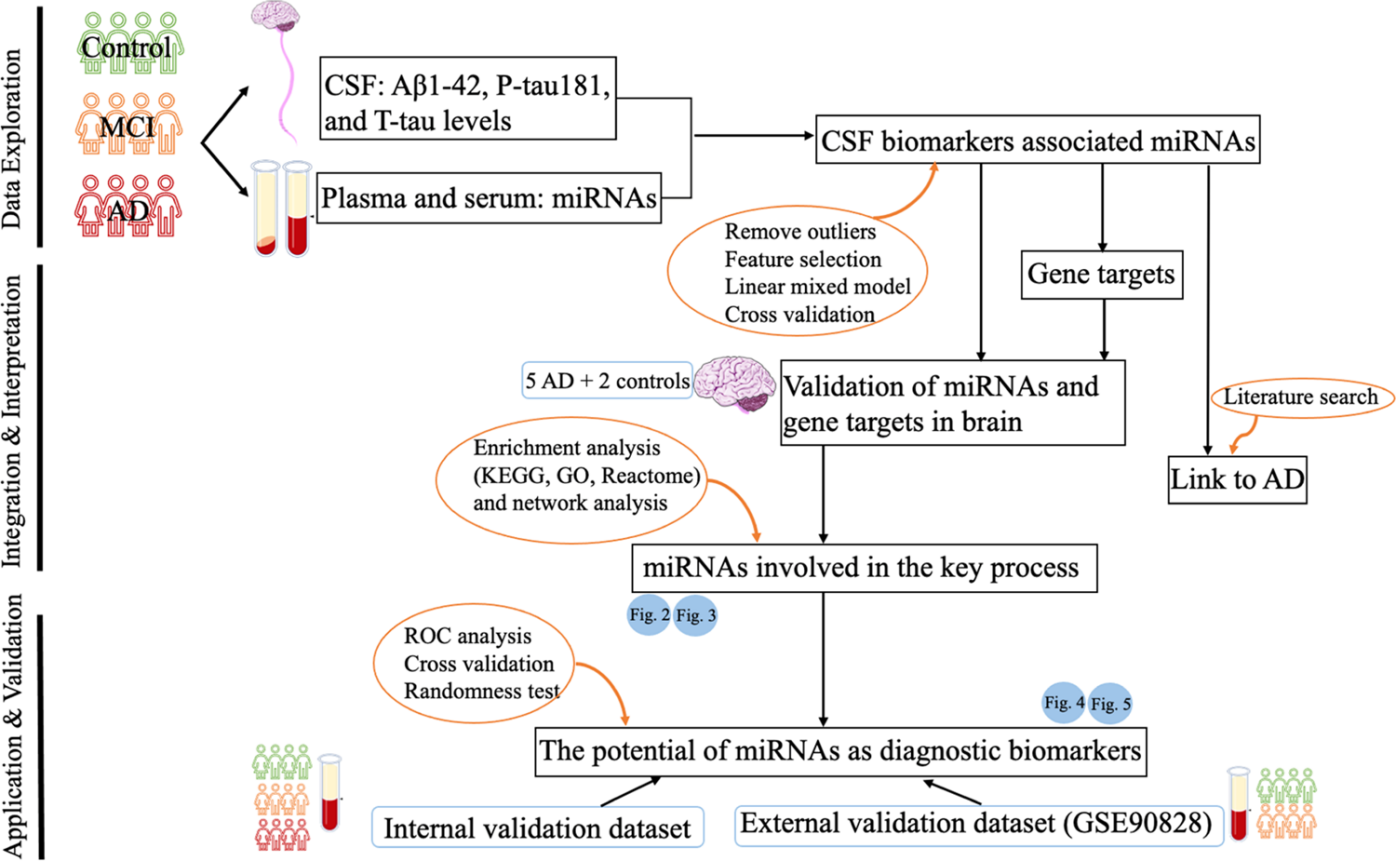
Workflow of this study. The miRNA sequencing data from plasma and serum samples of 112 participants including 28 Alzheimer’s disease (AD), 63 mild cognitive impairment (MCI), and 21 controls were used to identify cerebrospinal fluid (CSF) biomarkers associated miRNAs, and all associations were independent by potential confounders and cross-validated. The gene targets of these miRNAs were retrieved, and expression of both miRNAs and genes was confirmed in the human brain using brain tissue. Moreover, these miRNAs were considered relevant to AD based on the literature. The potential functions of these miRNAs were analyzed using over-representation analysis including the Kyoto Encyclopedia of Genes and Genomes (KEGG), Gene Ontology (GO), and Reactome. Pathways or biological processes present in the results of the KEGG, Reactome, and GO terms were considered the key pathways; using miRNAs and gene targets involved in the key biological process, we constructed a network. The potential of these miRNAs as biomarkers was explored using receiver operating characteristic (ROC) analysis, cross-validation, and randomness test. Both internal and external validation datasets were used.

In our study, we employed a comprehensive approach to uncover the relationship between circulating miRNAs and CSF biomarkers in individuals with sporadic AD, MCI, and controls (Figure 1). First, we harnessed the power of Lasso feature selection, as illustrated in Figure S1, to pinpoint miRNAs that explain the variations in CSF biomarkers. Subsequently, we conducted a rigorous linear mixed model analysis, as depicted in Figure S1, to identify circulating miRNAs associated with these CSF biomarkers across both serum and plasma samples. Our pipeline, outlined in Figure 1, led us to the discovery of seven miRNAs associated with Aβ1–42 and six miRNAs associated with P-tau181 in plasma samples. Additionally, we identified nine Aβ1–42-associated miRNAs in the serum samples. Notably, our investigation revealed no miRNAs associated with T-tau in either plasma or serum samples. All of these associations, as visualized in Figure S1, underwent rigorous cross-validation.

Since these blood-circulating miRNAs correlated with the levels of the AD biomarkers Aβ1–42 and P-tau181 in CSF, we examined their relevance to AD according to the literature. As shown in Table 1, most of these miRNAs play a potential role in relevant biological mechanisms involved in the development and progress of AD. For instance, miR-181a is shown to regulate RyanR3, which mediates the release of calcium from the endoplasmic reticulum; the dysregulation of this process is linked to synaptic loss and impaired cognitive function in AD.^40^

**Table 1:** Potential Role of our CSF Biomarker-Associated miRNAs in AD or other Neurodegenerative Diseases Based on a Literature Search

Specimen: Plasma			
miRNAs	associated to (Aβ1–42 or P-tau181) in our analysis	potential role in AD or other neurodegenerative diseases	refs
miR-126–5p	Aβ1–42	regulates growth factor activities in neurons	(41)
miR-145-5p	Aβ1–42	targeting of TRIM2 mediates the apoptosis of retinal ganglion cells via the PI3K/AKT signaling pathway	(42)
miR-181a-2–3p	Aβ1–42	targets RyanR3, which mediates the release of calcium from the endoplasmic reticulum, and a dysregulation of this process has been described as linked to synaptic loss and impaired cognitive function in AD	(40)
miR-194–5p	Aβ1–42	accelerates apoptosis of Aβ1–42-transduced hippocampal neurons by inhibiting Nrn1 and decreasing PI3K/Akt signaling pathway activity	(43)
miR-3177–3p	Aβ1–42 and P-tau181		
miR-323a-3p	Aβ1–42	may regulate amyloid precursor protein expression under physiological and pathological conditions	(44)
miR-342–5p	Aβ1–42	decreases ankyrin G levels	(45)
miR-22–3p	P-tau181	regulates Aβ1–42 deposit by targeting mitogen-activated protein kinase 14	(46)
miR-30e-5p	P-tau181	regulates neuroinflammation by targeting Nlrp3	(47)
miR-340–3p	P-tau181	reduces the accumulation of Aβ through targeting BACE1	(48)
miR-374b-5p	P-tau181	deregulated lncRNA MAGI2-AS3 sponges miR-374b-5p attenuates Aβ induced neurotoxicity and neuroinflammation	(49)
miR-494–3p	P-tau181	reduces DJ-1 expression and exacerbates neurodegeneration	(50)

Specimen: Serum			
miRNAs	associated to (Aβ1–42 or P-tau181) in our analysis	potential role in AD or other neurodegenerative diseases	refs
miR-106–5p	Aβ1–42		
miR-133a-3p/133b	Aβ1–42	regulates the maturation and function of dopaminergic neurons via suppression of paired-like homeodomain 3	(51)
miR-143–3p	Aβ1–42	inhibits aberrant tau phosphorylation and amyloidogenic processing of amyloid precursor protein in AD	(52)
miR-148b-3p	Aβ1–42		
miR-20b-5p	Aβ1–42	aggravates neuronal apoptosis induced by Aβ via down-regulation of the Ras homologue family member C in AD	(53)
miR-3613–5p	Aβ1–42		
miR-369–3p	Aβ1–42	loss of miR-369 promotes tau phosphorylation in AD mice	(54)
miR-4433b-5p	Aβ1–42		
miR-486–3p	Aβ1–42	influences the neurotoxicity of a-Synuclein by targeting the SIRT2 gene	(55)

### CSF Biomarker-Associated miRNAs Might Be Involved in AD Development via PI3K/AKT Signaling

**Figure 2:**
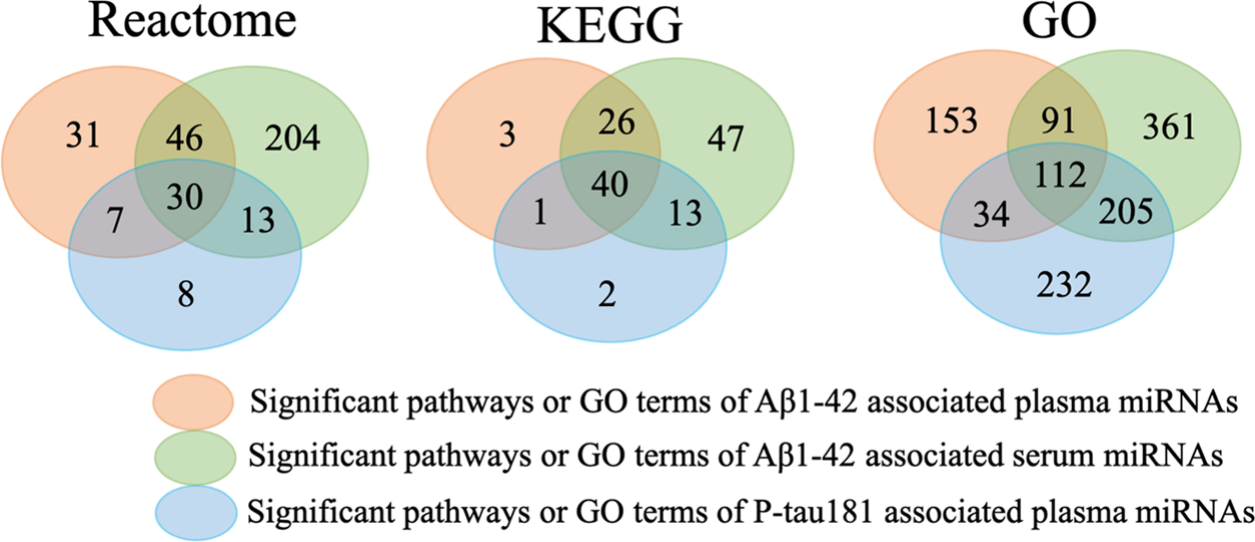
Overlap of significant pathways and Gene Ontology (GO) terms for these cerebrospinal fluid (CSF) biomarker-associated miRNAs. The numbers indicate the number of significant pathways or GO terms. For example, with enrichment analysis using Reactome, there are totally 114 (= 31 + 46 + 30 + 7) significant pathways for targets of amyloid β1–42 (Aβ1–42)-associated plasma miRNAs, and 30 pathways were common among phosphorylated tau181 (P-tau181)-associated plasma miRNAs and Aβ1–42-associated serum miRNAs. Similarly, 40 pathways were in common using the Kyoto Encyclopedia of Genes and Genomes (KEGG), and 112 GO terms were in common using GO.

We analyzed in depth how these circulating miRNAs could be involved in AD development in human brain tissues. First, we checked (see also the workflow in Figure 1) if these miRNAs and their gene targets were expressed using miRNA and mRNA sequencing data from brain tissues of five AD patients and two controls. Importantly, all these miRNAs and gene targets were expressed in human brain tissue of both the AD cases and controls. For further biological interpretation, we performed three different enrichment analyses, namely (1) Reactome, (2) KEGG, and (3) GO terms, for the brain-expressed gene targets of Aβ1–42-associated plasma miRNAs, of P-tau181-associated plasma miRNAs, and of Aβ1–42-associated serum miRNAs. In our KEGG pathway enrichment analysis, we pinpointed 70, 56, and 126 significantly enriched terms corresponding to Aβ1–42-associated plasma miRNAs, P-tau181-associated plasma miRNAs, and Aβ1–42-associated serum miRNAs, respectively (Table S1). Interestingly, we identified pathways relevant in AD pathology such as FOXO, cellular senescence, PI3K, MAPK, and EGFR. Similarly, for the Reactome, we identified 114, 58, and 293 significantly enriched terms corresponding to Aβ1–42-associated plasma miRNAs, P-tau181-associated plasma miRNAs, and Aβ1–42-associated serum miRNAs (Table S2), respectively, including AD-relevant pathways, e.g., PI3K, FOXO, cellular senescence, NOTCH1, etc. In our GO term enrichment analysis, we successfully identified a multitude of enriched terms for Aβ1–42-associated plasma miRNAs, P-tau181-associated plasma miRNAs, and Aβ1–42-associated serum miRNAs, specifically 390, 583, and 769 terms, respectively (Table S3). The convergence in pathway and GO term enrichment analyses across all three approaches indicates a potential shared involvement of these miRNAs in similar biological processes. However, the observation of unique pathways/GO terms exclusively enriched for Aβ1–42- or P-tau181-associated miRNAs suggests distinctive roles for these miRNAs in processes specific to the respective protein markers (Figure 2 and Table S4). We found that certain biological processes, such as transcription regulation, consistently exhibited enrichment across all approaches, including GO terms, Reactome, and KEGG, in all associated miRNA sets. Interestingly, among these, we identified the PI3K-Akt signaling pathway, which is one of the most important pathways in the development of AD.^56^ All our identified miRNAs target this pathway, which governs a multitude of biological processes, including cell proliferation, motility, growth, survival, and metabolic functions, while also acting to inhibit numerous neurotoxic mechanisms.^57^ More importantly, this pathway has been implicated in synaptic plasticity, learning, and memory processes.^58^ Moreover, Aβ and P-tau are also linked to PI3K/AKT signaling in previous publications^56,59^ (see also the Discussion section). In this analysis, the brain-expressed gene targets of Aβ1–42-associated plasma miRNAs, P-tau181-associated plasma miRNAs, and Aβ1–42-associated serum miRNAs were all enriched in this process.

**Figure 3:**
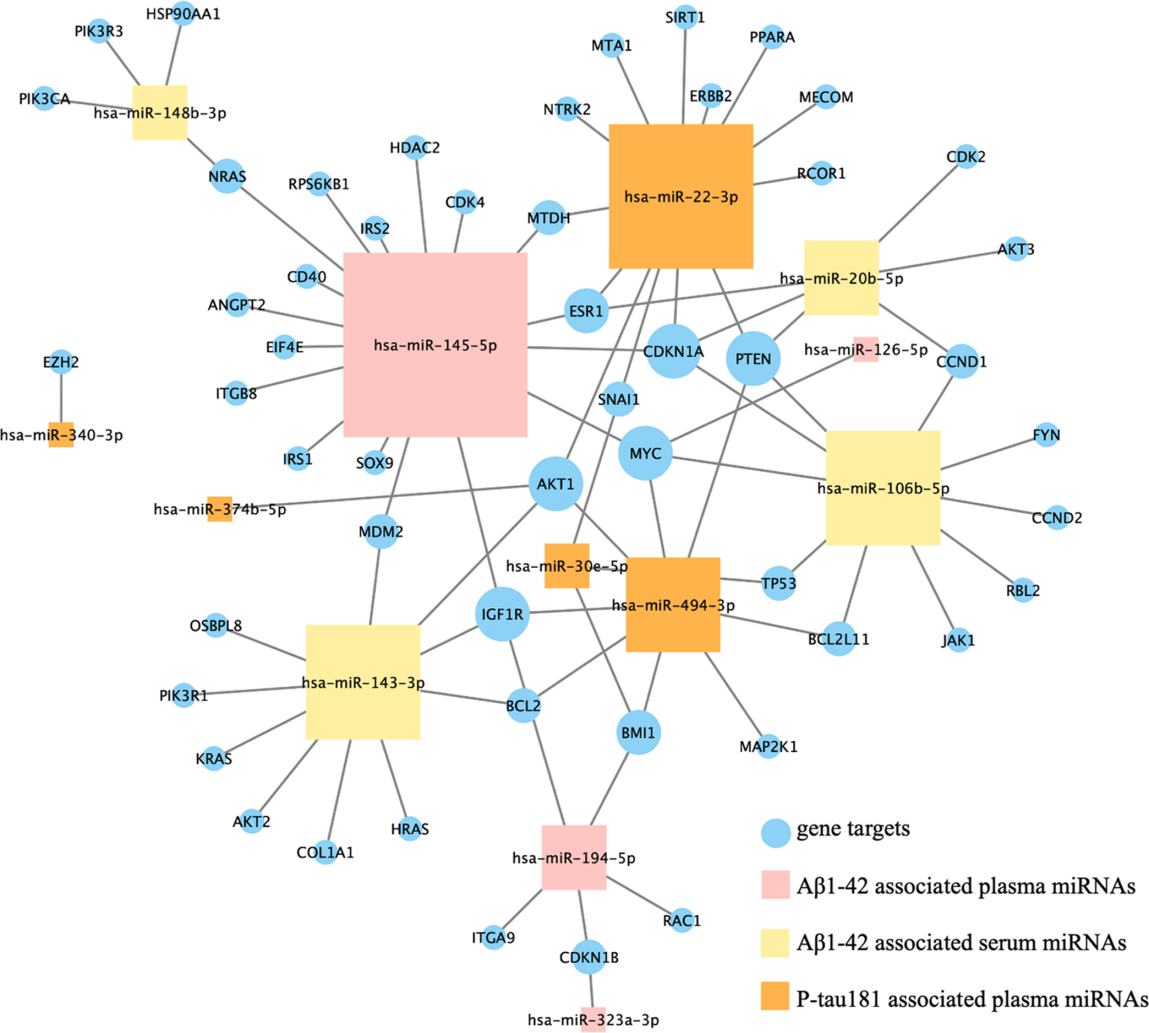
Interaction network for miRNAs and gene targets involved in the PI3K/AKT signaling pathway or process. The size of each sign was correlated to the number of lines connected to this sign; for example, miR-145-5p has the largest number of lines connected and therefore has the largest size, which means hub genes.

Using gene targets that were enriched in the PI3K/AKT signaling process and their corresponding miRNAs based on our sequencing data, we constructed an interaction network to search for the key miRNAs and gene targets involved in this process (Figure 3). The resulting hub miRNAs, including miR-145-5p, might possibly have important roles in AD (see below for further explanation). Similarly, the hub gene targets might also have important biological functions. For example, the gene PTEN functions as a hub gene, interacts with four miRNAs (Figure 3), and accordingly, it is shown that inhibition of PTEN rescued normal synaptic function and cognition in both cellular and animal models of AD.^60^

### Potential of Plasma miR-145-5p as a Diagnostic Biomarker in AD or MCI

The miRNA miR-145-5p was significantly related to CSF Aβ1–42 and was expressed in the studied human brain samples. Also, its brain-expressed gene targets are enriched in the AD-relevant PI3K/AKT signaling pathway or process. Next, we explored the potential of this miRNA as alternative biomarkers for AD or auxiliary biomarkers by combining this with the obtained MMSE scores, which can be obtained in a noninvasive and rather cheap way.

We included only the circulating miRNA data of plasma samples because more unique miRNAs were detected in plasma than in serum samples, even though more miRNA reads were detected in serum (Figure S2). In addition, for the circulating miRNAs which were both detected in plasma and serum samples, the levels of most miRNAs in plasma samples were significantly higher than in serum samples according to a *t* test (*p*-value <0.05) (Figure S2). Besides, based on the Pearson correlation coefficients, the correlations between the levels of miRNAs in plasma samples and serum samples were weak (−0.22 < *r* < 0.68) (Figures S3 and S4). Therefore, we considered only plasma miRNAs in the further analysis.

**Figure 4:**
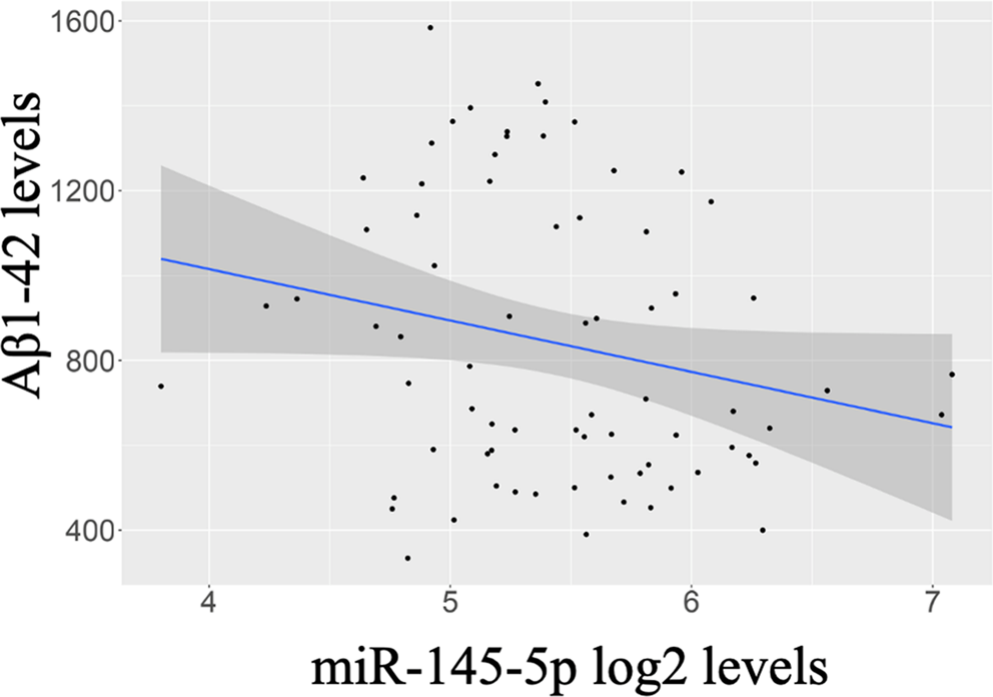
Associations among plasma miR-145-5p, amyloid β1–42 (Aβ1–42) loads in cerebrospinal fluid (CSF), and clinical disease state. The scatterplot shows the negative correlation between CSF Aβ1–42 concentrations (pg/mL) and plasma miR-145-5p levels, and this association is cross-validated as mentioned in the Methods.

**Figure 5:**
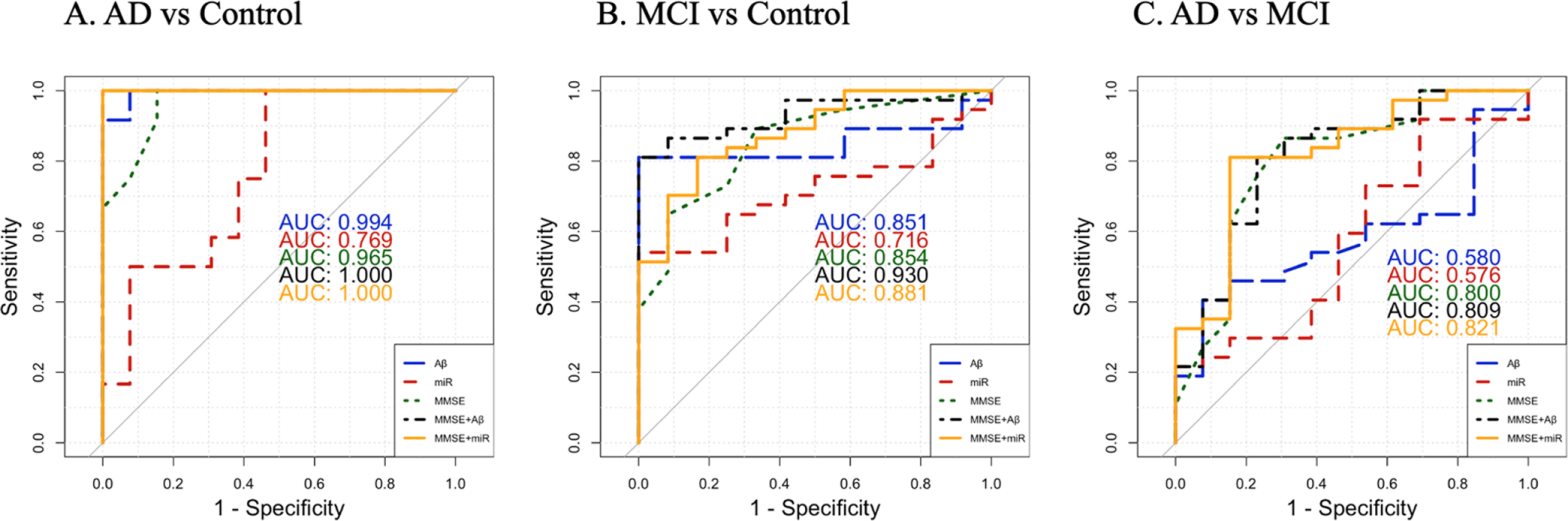
Area under the curve (AUC) scores of different biomarkers in predicting Alzheimer’s disease (AD) (A) and mild cognitive impairment (MCI) (B) from controls and predicting AD from MCI (C). In all three subplots, Aβ represents amyloid β1–42 (Aβ1–42) levels and miR represents miR-145-5p. In AD vs controls, miR-145-5p plus mini-mental state examination (MMSE) achieved an AUC score of 1.0, which is the same with Aβ1–42 plus MMSE, and improved MMSE’s performance, which is 0.97 AUC score. In MCI vs controls, miR-145-5p plus MMSE had a lower AUC score, which is 0.88 AUC score, still improved MMSE (AUC: 0.85). In AD vs MCI, even though the AUC score is lower than 0.6 using only miR-145-5p; this miRNA still has a comparable AUC score with Aβ1–42, and improved MMSE from 0.8 to 0.82 and performed better than Aβ1–42 did (0.81). The complete AUC scores for all different biomarkers are presented in Figures S6 and S7.

The CSF biomarkers associated with blood plasma-circulating miRNAs were shown to be good predictors for the AD continuum, with miR-145-5p emerging as a particularly strong predictor. In our analysis, we observed a negative association between CSF Aβ1–42 levels and plasma miR-145-5p levels (Figure 4). The levels of miR-145-5p were increased in the plasma of both AD and MCI cases with *p*-value <0.05. Notably, lower CSF Aβ1–42 levels were associated with worse outcomes (Methods section), supporting our findings. Using only one miRNA in classifying AD cases from controls, the area under the curve (AUC) scores of these Aβ1–42-associated and P-tau181-associated plasma miRNAs range from 0.53 to 0.77, among which miR-145-5p has the highest AUC score and performs better than most random selected miRNAs (Figure S5). Furthermore, adding the miR-145-5p level improved the prediction performance of the MRI biomarker (represented by normalized hippocampus volume) and MMSE score, even though there is no significant difference in two AUC, the AUC of MMSE plus miR-145-5p achieved 1(Figure 5A). Similarly, for MCI vs controls, miR-145-5p had the highest AUC score, which is 0.72, higher than that of most random miRNAs (Figure S5). Besides, there is no significant difference between the AUC of Aβ1–42 and miR-145-5p (*p* value >0.05). Additionally, it can improve the performance of the MRI biomarkers and MMSE score, surpassing even the performance of CSF biomarkers when combined with MRI biomarkers and MMSE scores (Figures 5B and S6). When distinguishing between AD and MCI cases, it is notably more challenging to differentiate AD cases from MCI cases even though some miRNAs exhibited superior AUC scores compared to those of CSF biomarkers for this purpose (Figure S7). While the miRNA miR-145-5p alone in predicting AD from MCI yields a lower AUC score (less than 0.6), it suggests results comparable to those of Aβ1–42 and even outperforms Aβ1–42 when combined with the MMSE score (Figure 5C).

### Performance of miR-145-5p Was Internally and Externally Validated

**Figure 6:**
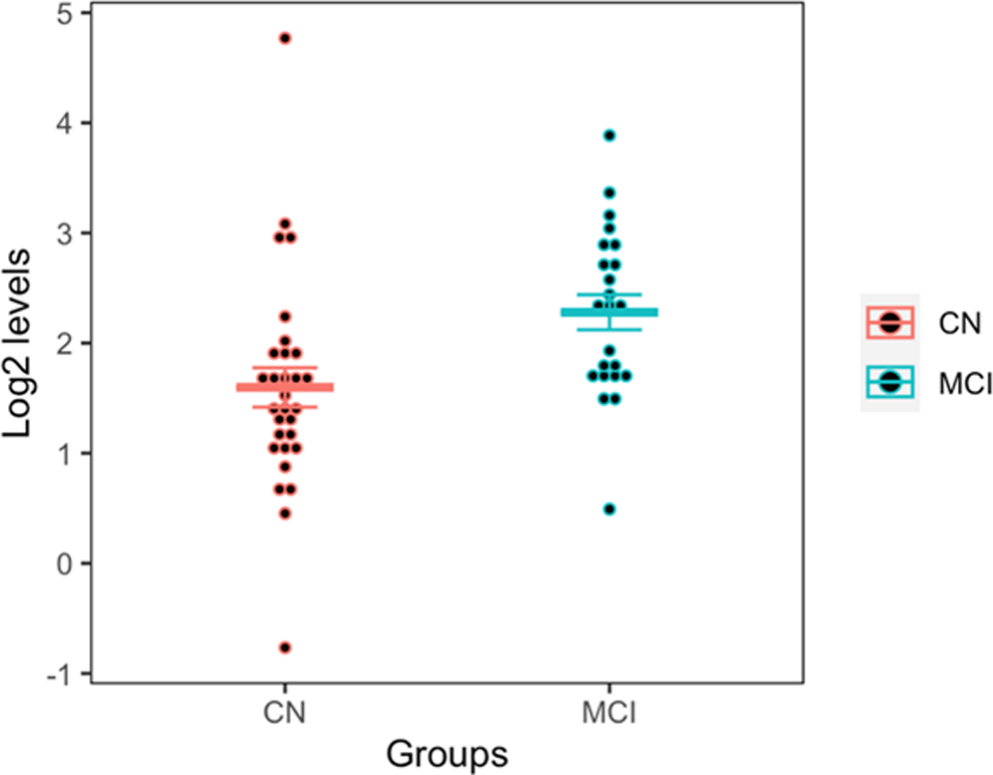
Plasma levels of miR-145 for both mild cognitive impairment (MCI) and control groups in the qPCR dataset GSE90828. Using the *t* test, the levels of miR-145 in the MCI group are significantly higher than those in the control group (*p* value <0.05).

To confirm the potential of miR-145-5p further, we used both an internal validation dataset (based on the sequencing data excluded in linear mixed model analysis due to the missing value in potential confounders) and an external validation dataset (based on the qPCR dataset GSE90828). For AD vs controls, the AUC score of miR-145-5p with the internal dataset (six AD cases and six controls) is 0.67. For MCI vs controls, the AUC score of miR-145-5p in the internal dataset (eight MCI cases and six controls) is 0.65, the AUC score of miR-145-5p with the external dataset (23 MCI cases and 30 controls) is 0.76. Additionally, Figure 6 illustrates the levels of this miRNA for both the MCI and control groups in the qPCR dataset. The plasma levels of miR-145 in MCI samples are significantly higher than those in control groups (*p* value <0.05) in this external dataset, and this finding is consistent with the results observed in our own dataset, as described above.

## Discussion

Extensive screening in populations using noninvasive and relatively easy-to-obtain biomarkers for sporadic AD is important, and circulating miRNAs might be a potential candidate. In this study, blood plasma- and serum-circulating miRNAs were related to established CSF biomarkers, which are the most commonly used early and precise diagnostic biomarkers for AD so far. The identified circulating miRNAs were estimated to link to AD-relevant processes such as the PI3K/AKT signaling process, which plays an important role in AD development. Plasma miR-145-5p demonstrated the best performance in predicting the AD continuum. Furthermore, it enhanced the predictive performance of MMSE scores and outperformed CSF Aβ1–42 when combined with MMSE scores and MRI biomarkers.

Previous studies have attempted to relate circulating miRNAs to CSF biomarkers before. For example, differentially expressed serum miRNAs in AD patients versus controls were used to construct a panel of seven miRNAs to predict P-tau181/Aβ1–42 in CSF.^61^ Even with different analysis approaches, there is one common serum miRNA (miR-143–3p), and it was shown that inhibition of this miRNA might promote neuronal survival according to an AD cell model.^62^ In another study, a significant association among miR-27a-3p, miR-27b-3p, and miR-324–5p and Aβ loads among cognitively normal Aβ-positive, MCI, and AD participants was identified.^31^ No overlapping miRNAs were found, possibly due to different populations, disease stages, and study design. However, these miRNAs also target the PI3K/AKT signaling pathway, which is consistent with the process identified in our study. Furthermore, it has been suggested that plasma miRNA levels change before the onset of symptoms and vary dynamically with disease progression.^31^

There has been increasing evidence suggesting that the PI3K/AKT pathway plays a key role in the pathophysiology of AD.^56,59,63^ This pathway governs a multitude of biological processes, including cell proliferation, motility, growth, survival, and metabolic functions, while also acting to inhibit numerous neurotoxic mechanisms.^57^ The PI3K/AKT pathway can be activated by oxidative stress, which is an inseparable part of AD pathogenesis.^56^ Further, the modulation of PI3K/Akt signaling has been suggested as a potential therapeutic target in AD.^56,63^ As an intracellular signaling pathway, PI3K/AKT can regulate amyloid-induced neurotoxicity and mediate the survival of neurons via different substrates such as glycogen synthase kinase-3β (GSK-3β).^63^ Various targets in the downstream of this pathway are related to the occurrence and development of AD. For instance, the increase of GSK-3β activity is related to the increase of Aβ production and deposition, hyperphosphorylation of tau, and the formation of neurofibrillary tangles.^63^ Additionally, PI3K/AKT signaling can also regulate neuronal synaptic plasticity and memory processes.^58,63,64^ In this study, the identified circulating miRNAs all target this pathway, which suggested a role for these miRNAs in AD development and progression.

Recently, the miR-145-5p level and PI3K/AKT signaling pathway have also attracted increased attention in the study of other diseases^65−67^ such as cancer and type 2 diabetes. There is mounting evidence suggestion that type 2 diabetes is associated with cognitive dysfunction, and individuals with diabetes have been reported to have an elevated risk of developing various forms of dementia, including AD.^68^ MiR-145-5p has also been reported to be downregulated in CSF of AD patients; however, no consistent result has been found across cohorts.^69^ In our study, we suggested the potential of miR-145-5p as a diagnostic biomarker for AD via ROC analysis and validation datasets and its largest interactions within the PI3K/AKT signaling process (Figure 3). PI3K/AKT has also been shown to be overactivated in many human cancers. Interestingly, according to a population-based study, there is inverse occurrence of cancer and AD, showing that elderly persons with cancer have a reduced risk of AD dementia and vice versa.^70^ Furthermore, the deregulation of the cell cycle via this pathway activation has been considered the answer for the overlapped pathogenesis between AD and cancer with a diverse destiny.^66^ Also, miR-145-5p has been proposed as a potential biomarker for many diseases including type 2 diabetes^65^ and cancers,^71,72^ and the PI3K/AKT signaling pathway has been a common mechanism connecting different diseases such as AD and cancer^66^ and AD and type 2 diabetes.^67^ In our study, when exploring the potential of CSF biomarker-associated miRNAs as biomarkers, we tested all possible combinations of these miRNAs as classifiers. The combination of these miRNAs with the optimal AUC score did not improve the performance of miR-145-5p in validation datasets. Additionally, miR-145 has been reported to ameliorate oxidative stress in retinal endothelial cells^73^ and cardiomyocytes,^74^ while promoting oxidative stress in microglias.^75^ This aspect shows the potential role of miR-145-5p in neurodegenerative diseases, which are partly characterized by oxidative stress. To show the specific potential of miR-145-5p in AD, we combined the MMSE score with miR-145-5p, a combination of biomarkers and symptoms, resulting in an outstanding prediction of MCI and AD cases (Figure 5). This kind of approach is promising in screening of the general population, since it is cheaper, minimally invasive, and easily monitored. Furthermore, future mechanistic studies focusing on the interactions between miR-145-5p and the mRNAs involved in the PI3K/AKT signaling pathway would be vital to understand their role in disease development.

Blood plasma samples were used as specimens for miRNA detection in our study. Even though both plasma and serum samples were used to identify miRNA biomarkers,^76,77^ a qualitative study on rodents and humans using high-throughput miRNA sequencing suggested to use plasma samples due to the fewer reads with length corresponding to non-miRNA sequences observed in plasma than in serum.^78^ In another study, plasma is suggested to be the sample of choice in studying circulating miRNA because RNAs including miRNAs released by cells during the coagulation process may change the true repertoire of circulating miRNAs.^79^ Accordingly, in our analysis, the correlations between plasma miRNAs and serum miRNAs were weak (Figures S3 and S4). Besides, there were more miRNA reads in serum, while more unique miRNAs and a significantly higher level of common miRNAs were detected in plasma than in serum (Figure S2). Therefore, one of the reasons for the variance of miRNAs in plasma and serum might be that additional miRNA was released during the coagulation process.^79^ Additionally, further studies for the impact of sample preparation, such as the influence of the coagulation process on the detection of circulating miRNAs in serum, are needed to better understand the differences between plasma miRNAs and serum miRNAs.

There may be some possible limitations in this study. The larger sample size and a prospective longitudinal study are needed for validation, even with our rigorous and comprehensive analytical process. Especially, it is crucial to validate our findings further in diverse AD cohorts, taking into account characteristics ideal for validation, such as demographic diversity, disease severity, and genetic variations within the cohorts. Additionally, potential challenges, including variations in sample collection methods and diagnostic criteria, should be addressed. Also, in our study, population ages were significantly lower in the control than in AD and MCI cases. Considering that age is an important risk factor for AD, a follow-up study including age-matched control subjects in the future is necessary. Besides, 9 serum samples and 23 plasma samples were removed due to low sequence coverage. We checked the correlation between storage time and sequence depths, but no significant correlation was found; therefore, the reason for the low sequence depths of these samples remains unknown. Even though p-Tau181 is commonly utilized as a validated biomarker in routine biochemical assessments, there is still potential benefit in investigating the association between p-Tau217 or p-Tau231 and miRNAs. Despite the fact that lower sequence depths were also used in previous publications,^80,81^ future studies with higher sequence coverage are preferable. The MMSE test is a measure for global cognition, and while it is only used as a screening test instead of diagnostic purposes, its accuracy for cognitive function measurement is still debated. Besides, it would be valuable to validate the combination of miR-145 and the MMSE score in the external validation dataset. Lastly, the plasma miR-145-5p as a potential biomarker was only externally validated in MCI due to AD samples but could not be validated in AD in other patient samples due to limited available datasets and samples. Although MCI participants in our study and previously published dataset were either MCI due to AD or amnestic MCI, which was the type of MCI most likely to develop AD, further external validation is essential in additional circulating miRNA datasets obtained from AD patients. Even though with limitations regarding sample size, linear mixed model is still beneficial for this study to incorporate more clinical information, as only a few miRNAs were identified as differentially expressed in AD or MCI (see Figure S8).

To summarize, even though further validation is needed, the identified plasma miRNAs show promise as potential biomarkers for the AD continuum in the general population. This is particularly significant as obtaining these samples is less invasive than obtaining CSF samples and more cost-effective than measuring biomarkers through PET or MRI. Our study employed a linear mixed model to explore the association between a broad range of circulating miRNAs and CSF biomarkers. This approach maximizes the correlation between circulating miRNAs and CSF biomarkers within the context of Alzheimer’s and incorporates the clinical information. In addition, brain tissues were utilized to validate the identified miRNAs and their targets. As a result, we identified new miRNAs related to CSF biomarkers. Among them, miR-145-5p stands out as it exhibits an association with CSF biomarkers and demonstrates good predictive power on its own or when combined with MMSE scores in AD or MCI due to AD. To the best of our knowledge, this study is the first to suggest the combination of miR-145-5p and the MMSE score as a promising screening tool for AD in the general population. Furthermore, in-depth interpretation showed that this miRNA interacts with the PI3K/AKT signaling pathway, indicating the biological relevance in the disease development.

## Methods

### Samples

The study population (Table 2) comprises 112 subjects, which included 28 dementia due to AD cases, 63 MCI due to AD cases (sporadic), and 21 cognitively healthy controls. Blood was drawn in standard vacutainer EDTA blood tubes from Becton Dickinson (BD) and within 2 h of collection centrifuged for 10 min at 1600*g* at 4 °C, and the plasma layer was pipetted into new tubes. After collection of the whole blood in a standard BD vacutainer, the blood was allowed to clot by leaving it undisturbed at room temperature. The clot was removed by centrifuging for 10 min at 1600*g* at 4°. Both 105 plasma samples and 112 serum samples of these subjects were collected and stored at −80 °C by the Institute Bor*n*-Bunge biobank, in Antwerp (Belgium). These samples were collected from March 14, 2013 to July 4, 2017. In the context of the EU Interreg project Memories (www.herinneringen.eu), these samples were sent on dry ice to Maastricht University, Maastricht (The Netherlands). Patient classification was effectuated in compliance with the NIA-AA criteria for “MCI due to AD” and “Dementia due to AD”. Only patients exhibiting an AD CSF biomarker profile, characterized by decreased values of Aβ1–42 (or a reduced Aβ1–42/Aβ1–40 ratio) and elevated T-tau or P-tau181 levels, will be eligible for inclusion in the study. Controls were cognitively healthy, having no cognitive complaints as objectively determined by means of a neuropsychological examination. Controls were recruited among spouses of patients and through advertisement to the general public. The exclusion criteria for the total population included brain tumors, large cerebral infarction/bleeding, other neurodegenerative diseases, severe head trauma, epilepsy, brain infections, severe depression, and contraindications for lumbar puncture or MRI, such as coagulation disorders, the use of anticoagulant medication, anemia, uncontrolled hypertension, and the presence of pacemakers. This study was conducted in 2022; the authors had access to information that could identify individual participants after data collection.

**Table 2:** Study Population Data Including Gender, Age, Years of Education, Aβ1-42, P-tau181, and T-tau levels, MMSE Score, MRI Biomarker (Hippocampus Volume), and Medication^a^,^b^

	total population (*n* = 112)	AD (*n* = 28)	MCI (*n* = 63)	controls (*n* = 21)	P1 (AD vs controls)	P2 (MCI vs controls)	P3 (AD vs MCI)
male	58 (51.8%)	18 (64.3%)	29 (46.0%)	11 (52.4%)			
age (years)	70.4 ± 8.4	72.6 ± 7.1	72.2 ± 7.8	62.1 ± 7.2	<0.001	<0.001	0.8
education (years)	18.2 ± 3.6	17.8 ± 4.3	18.0 ± 3.4	19.1 ± 2.7	0.17	0.13	0.76
Aβ1–42 level (pg/mL)	797.4 ± 346.7	571.8 ± 182.4	759.7 ± 332.5	1218.6 ± 135.5	<0.001	<0.001	<0.05
P-tau181 level (pg/mL)	71.5 ± 32.0	84.4 ± 34.3	75.5 ± 30.3	42.1 ± 8.8	<0.001	<0.001	0.28
T-tau (pg/mL)	461.6 ± 275.8	639.2 ± 307.9	470.6 ± 239.2	196.8 ± 42.1	<0.001	<0.001	<0.05
MMSE score	24.2 ± 5.3	18.2 ± 6.2	25.2 ± 3.0	28.9 ± 1.5	<0.001	<0.001	<0.001
hippocampus volume (mL)	8.1 ± 1.2	7.4 ± 0.8	8.1 ± 1.2	9.1 ± 1.0	<0.001	<0.05	<0.05
SNRI	19 (17.0%)	5 (17.9%)	12 (19.0%)	2 (9.5%)			
PPI	11 (9.8%)	3 (10.7%)	8 (12.7%)	0 (0%)			

a SNRI (serotonin–norepinephrine reuptake inhibitor) is used for depression such as Venlafaxine. PPI (proton pump inhibitor) is used for gastroesophageal pathology and symptoms, such as Pantomed. These rows show the number and percentage using this medication. Age, Aβ1-42 level, P-tau181 level, T-tau level, MMSE score, and hippocampus volume were significantly different in both AD vs control group and MCI vs control group; thereinto, Aβ1-42 level, T-tau level, MMSE score, and hippocampal volume were significantly different in AD vs the MCI group.

b The description of the variables is presented as mean ± SD or *n* (%).

Additionally, brain tissues from five sporadic AD cases and two age-matched nondementia controls were included in this study to confirm the expression of both our miRNAs and their gene targets in the human brain. These samples also came from the Institute Bor*n*-Bunge biobank and had a volume of 1 cm3 that was dissected with the same protocol^82^ and snap-frozen within 6 h after death. The detailed information on these samples is described in previous publications.^83,84^

### CSF Biomarkers, MRI Biomarker, and MMSE Score

Lumbar puncture, subsequent CSF sampling, and further handling (including storage at −80 °C until analysis) were performed according to a previously described standard protocol.^85^ To perform the Aβ1–42, T-tau, and P-tau181 CSF biomarker analysis, a commercially available single parameter ELISA kit (INNOTEST, Fujiribio Europe, Ghent, Belgium) was used. Further details regarding the complete CSF analysis procedure have been published.^86^ The following present clinical cutoffs were used: normal values are Aβ1–42 > 638.5 pg/mL, P-tau181 < 56.5 pg/mL, and T-tau <296.5 pg/mL. MRI was performed on each subject on a 3T whole body scanner with a 32-channel head coil (Siemens Trio/PrismaFit, Erlangen, Germany). To obtain 176 axial slices (without a slice gap) and 1.0 mm nominal isotropic resolution (FOV = 192 mm × 256 mm), the 3D MP-RAGE (TR/TE = 2200:2.45 ms) was used. A 3D T1w MR sequence was obtained from all participants. An automated brain imaging morphometry analysis was performed by icobrain dm (v.4.4.0), which is thoroughly described elsewhere.^87^ In this study, the normalized hippocampus volume was used. A neuropsychological test battery was performed on each participant, including an MMSE score (0/30), which was taken less than three months before or after MRI.

### Small RNA Sequencing

Plasma and serum RNAs were isolated using the miRNeasy serum/plasma Kit. The tRNA/YRNA depletion protocol was used. Libraries were prepared for sequencing with the automated Gel-Free option in NEXTFLEX Small RNA-Seq Kit v3. For both plasma and serum samples, 200 ul of RNA was used for sequencing. The Illumina NovaSeq 6000 was used to sequence all samples (112 serum samples and 105 plasma samples). Samples were sequenced with all Phred scores above 31.7 and an average Phred score of 35.

After quality control using FastQC (version 0.11.7) and trimming of adapters and random bases using Cutadapt (version 1.8.1), data were processed with miRge2^88^ with the latest release of miRbase. The resulting expression matrix was analyzed using open-source software R (version 4.1.0). Due to insufficient sequencing coverage (sequence depths <0.1 millions reads), 9 serum samples and 23 plasma samples were removed in the subsequent analysis. Besides, miRNAs were excluded when measured in less than 75% of samples. Data normalization and differential expression analysis were conducted separately for plasma and serum samples using the DESeq2 package.^89^ Finally, the expression level of 241 miRNAs in 82 plasma samples (19 AD cases, 45 MCI cases, and 18 controls) and the expression level of 210 miRNAs in 103 serum samples (26 AD cases, 58 MCI cases, and 19 controls) were used in the following analysis.

### Statistical Analysis

The analysis workflow is shown in Figure 1. Data for plasma and serum samples were processed using the same procedures. For each CSF biomarker (Aβ1–42, P-tau181, and T-tau), we removed the outliers defined by 1.5 IQR (interquartile range). The normality of miRNA expression data for both plasma and serum samples was checked using the plotDensities function in R (Supporting Figure S9). Then, we conducted feature selection to get the miRNAs which are relevant to variation among Aβ1–42, P-tau181, or T-tau levels using the least absolute shrinkage and selection operator (Lasso)^90^ by the glmnet package in R. For plasma samples, 241 miRNAs were included, among which 8 miRNAs were recognized as explanatory variables for the Aβ1–42 level and 11 miRNAs were recognized as explanatory variables for the P-tau181 level. For serum samples, 210 miRNAs were included, among which 27 miRNAs were recognized as explanatory variables for the Aβ1–42 level and no miRNAs were recognized as explanatory variables for the P-tau181 level. For both plasma and serum samples, no confounders were considered at this feature selection step, and no miRNAs were recognized as explanatory variables for the T-tau level. The result for Lasso is presented in Figure S1. To avoid overfitting the model, cross-validation was used. Next, a linear mixed model was used to identify CSF Aβ1–42, P-tau181, or T-tau level-associated miRNAs among the above miRNAs, and potential confounders including age, gender, education, and medications were corrected. Furthermore, the technical variations introduced by the different batches of RNA isolation and library preparation were added as random effects. CSF biomarkers’ levels were defined as the variable of interest in three models separately. Resulting *p*-values were controlled by a false discovery rate (FDR) at 10%. Correction of confounders was included in the model if significant miRNAs could be observed in the association with a potential confounder. For numeric variables, this was determined by plotting *p*-values in quantile–quantile plots (QQ plots) using a linear model; for categorical variables, this was determined by differential expression analysis with the DESeq2 package; finally, we obtained the models used in plasma samples and serum samples (Supporting Figure S10). In the final linear mixed models, for plasma samples, age, gender, education, and medication of PPI and SNRI were used as confounders, and the year of sample collection and the date of RNA isolation and library were used as random effects. For serum samples, age, gender, education, and medication of SNRI were used as confounders, and the year of sample collection and the date of RNA isolation and the library were used as random effects. All associations in the linear mixed model were cross-validated by leave-one-out cross-validation.

The function *t* test in R was used to compare plasma miRNAs and serum miRNAs. To analyze the correlation between plasma miRNAs and serum miRNAs, Pearson correlation coefficients with the function cor and visualization with the function corplot from the dplyr package were used.

We conducted ROC analysis only for plasma samples in this study to evaluate the performance of these CSF biomarker-associated miRNAs as a classifier in AD cases vs controls, in MCI cases vs controls, and in AD cases vs MCI cases. Upon combining our miRNAs with other clinical screening or diagnostic biomarkers such as the MMSE score and MRI biomarkers, we used logistic regression by the stat package in R to predict the possibility of samples as cases. Cross-validation and the random test were performed in all ROC analyses. The Delong test was used in comparing two AUC values; only with a p value of <0.05, two AUC were recognized as significantly different. Part of the plots were formed by ggplot.^91^Figure 1 is partly generated using Servier Medical Art, provided by Servier, licensed under a Creative Commons Attribution 3.0 Unported License (https://creativecommons.org/licenses/by/3.0/).

### Over-representation Analysis and Network Analysis

From the experimentally validated miRNA-target interaction database miRTarBase (release 9.0),^92^ all gene targets of CSF biomarker-associated miRNAs were retrieved; only interactions determined by the “strong evidence” validation method including reporter assay, Western blot, Northern blot, and qPCR were included. Brain-expressed gene targets of brain-expressed miRNAs were analyzed for their over-representation in the biological process. Since many relevant pathway resources or GO terms have been reported and each one focused on different aspects, to obtain more reliable and well-found biological process, in this study, three repositories including the KEGG (Kyoto Encyclopedia of Genes and Genomes), GO (Gene Ontology), and Reactome were used in enrichment analysis. For KEGG pathways and GO terms, the clusterProfiler package in R was used.^93^ Pathways or GO terms with a p-value of <0.01 (FDR-corrected) were considered significantly overrepresented. All genes present in the key biological process with their miRNA interactions based on miRTarBase were exported from R to Cytoscape (version 3.9.1) and visualized via the network.

### Internal Validation

In the linear mixed model (see the Statistical Analysis section), 20 plasma samples (six AD cases, eight MCI cases, and six controls) were removed due to missing values in the explanatory variables. These samples were used as an internal validation dataset in the ROC analysis.

### External Validation

Publicly available miRNA RT-qPCR data GSE90828 were used as an external validation dataset to validate the performance of miRNAs. We used the keywords “Alzheimer’s disease” or “AD” plus “microRNAs” or “miRNAs” or “Noncoding RNA” plus “plasma” in the GEO datasets to search for available datasets and selected only *Homo sapiens*. Based on the sample size, GSE90828 which is an RT-qPCR dataset comprising 745 miRNAs from plasma samples of 23 MCI patients and 30 age-matched controls was used as external validation.^39^

## Data Availability

The miRNA sequencing data of 112 subjects have been submitted to the NCBI GEO repository with the accession number GSE215789 (https://www.ncbi.nlm.nih.gov/geo/query/acc.cgi?acc=GSE215789). The clinical information is not publicly available due to restrictions imposed by the Belgian/Dutch legislation on the protection of personal data. Request to access the data should be directed to Sebastian Engelborghs, sebastiaan.engelborghs@uantwerpen.be. The RT-qPCR dataset used in the external validation is a publicly available dataset that can be found at the GEO with GSE90828, doi: 10.1186/s40364–016–0076–1 (2016).

## Supporting Information Available

Results of feature selection, linear mixed model, and cross-validation, comparison between plasma miRNAs and serum miRNAs, AUC scores regarding different biomarkers in predicting AD and MCI, and volcano plots for differentially expressed miRNAs in serum and plasma samples regarding different comparing groups (PDF)
Results for enrichment analysis with GO, KEGG, and Reactome (XLSX)

## Notes

This study conformed to the Declaration of Helsinki. All participating subjects provided a written informed consent. The ethics committee review board has approved the research protocol.

## Abbreviations

AD: Alzheimer’s disease
MCI: mild cognitive Impairment
CSF: cerebrospinal fluid
Aβ1–42: amyloid β1–42
T-tau: total tau
P-tau181: phosphorylated tau181
miRNA: microRNA
mRNA: mRNA
DEmiRNAs: differentially expressed miRNAs
MMSE: mini-mental state examination
MRI: magnetic resonance imaging
Lasso: least absolute shrinkage and selection operator
KEGG: Kyoto Encyclopedia of Genes and Genomes
GO: gene ontology
FDR: false discovery rate
ROC: receiver operating characteristic
AUC: area under the curve
PET: positron emission tomography
NIA-AA: National Institute on Aging-Alzheimer’s Association
SNRI: serotonin–norepinephrine reuptake inhibitor
PPI: proton pump inhibitor
