# Supplementary material for: Beyond CSF and Neuroimaging Assessment: Evaluating Plasma miR-145-5p as a Potential Biomarker for Mild Cognitive Impairment and Alzheimer’s Disease
Source: ACS Chem Neurosci. 2024 Feb 26;15(5):1042–54. doi: 10.1021/acschemneuro.3c00740 (PMC10921410; doi:10.1021/acschemneuro.3c00740)
Supplement: Supplementary file 1 — cn3c00740_si_001.pdf [file cn3c00740_si_001.pdf]

Supporting information

Beyond CSF and neuroimaging assessment: Evaluating plasma miR-145-5p as a potential biomarker for mild cognitive impairment and Alzheimer's disease

Qingfeng Wen[a,b], Mandy Melissa Jane Wittens[c,d,e], Sebastiaan Engelborghs[c,d,e], Marcel HM van Herwijnen[a], Maria Tsamou[f], Erwin Roggen[f], Bert Smeets[a,b], Julian Krauskopf[a,1], and Jacco Jan Briedé[a,b,1]

a. Department of Toxicogenomics, Maastricht University, Universiteitssingel 50, 6229 ER Maastricht, the Netherlands

b. MHeNS, School for Mental Health and Neuroscience, Maastricht University, Universiteitssingel 50, 6229 ER Maastricht, the Netherlands

c. Department of Biomedical Sciences, Institute Born-Bunge, University of Antwerp, Universiteitsplein 1, BE-2610 Antwerpen, Belgium

d. Neuroprotection and Neuromodulation (NEUR), Center for Neurosciences (C4N), Vrije Universiteit Brussel (VUB), Laarbeeklaan 103, 1090 Brussel, Belgium

e. Department of Neurology, Universitair Ziekenhuis Brussel (UZ Brussel), Laarbeeklaan 101, 1090 Brussel, Belgium

f. ToxGenSolutions (TGS), Maastricht, The Netherlands.

Correspondence to QW (email: q.wen@maastrichtuniversity.nl)

[1] JK and JJB share senior authorship

Figure S1. The association between cerebrospinal fluid (CSF) biomarkers (amyloid β1-42 (Aβ1-42) and phosphorylated tau181 (P-tau181)) levels and miRNAs levels. First, CSF biomarkers relevant miRNAs were selected using least absolute shrinkage and selection operator (Lasso), then a linear mixed model was used to identified significantly associated miRNAs, finally the coefficients of these significant CSF biomarkers associated miRNAs were cross validated. A. Plasma samples: seven miRNAs are recognized as Aβ1-42 associated miRNAs with two miRNAs negatively associated and five miRNAs positively associated (I), six miRNAs are recognized as P-tau181 associated miRNAs with three miRNAs negatively associated and three miRNAs positively associated (II). All significant miRNAs were cross validated. B. Serum samples: nine miRNAs are recognized as Aβ1-42 associated miRNAs with six miRNAs negatively associated and three miRNAs positively associated (I), no miRNAs are recognized as P-tau181 associated miRNAs (II). All significant miRNAs were cross validated.

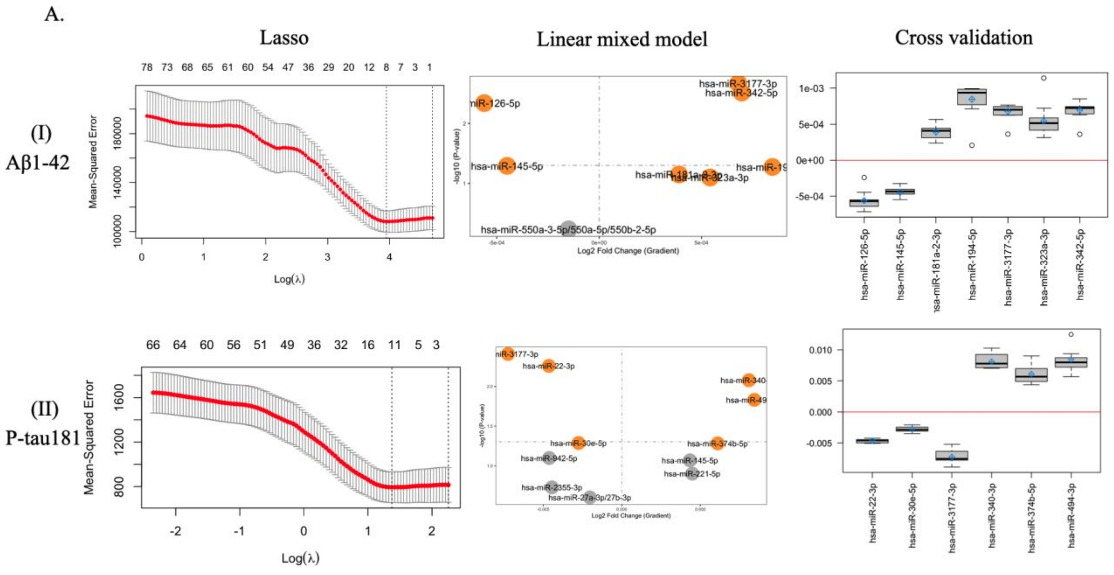
A.
Lasso
Linear mixed model
Cross validation
(I)
Aβ1-42
(II)
P-tau181
Mean-Squared Error
Log(λ)
Log2 Fold Change (Gradient)
-log10 (P-value)
hsa-miR-3177-3p
hsa-miR-342-5p
hsa-miR-145-5p
hsa-miR-323a-3p
hsa-miR-550a-3-5p/550a-5p/550b-2-5p
hsa-miR-126-5p
hsa-miR-181a-2-3p
hsa-miR-194-5p
hsa-miR-22-3p
hsa-miR-30e-5p
hsa-miR-942-5p
hsa-miR-2355-3p
hsa-miR-27a-3p/27b-3p
hsa-miR-374b-5p
hsa-miR-221-5p
hsa-miR-340-3p
hsa-miR-494-3p

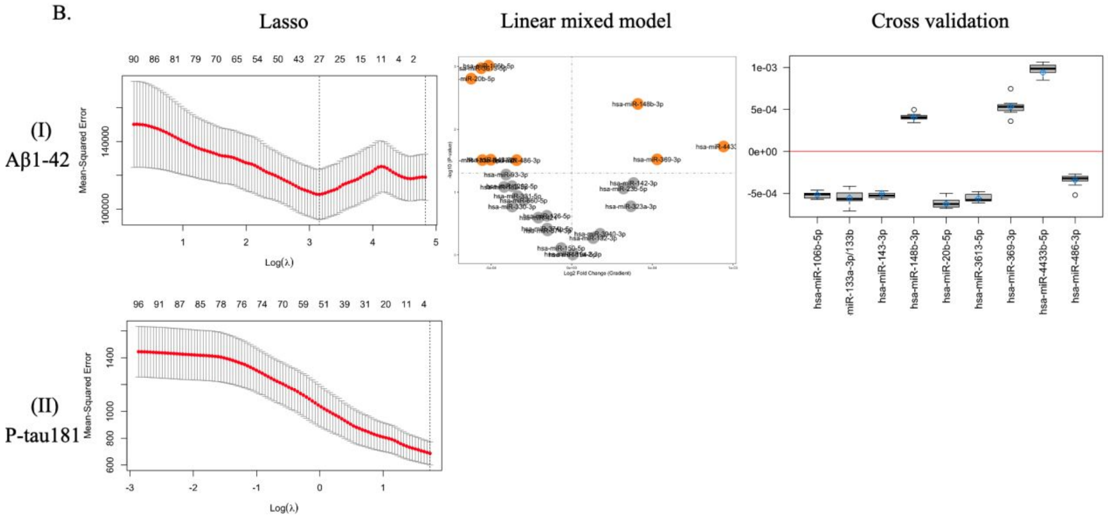

Figure S2. The comparison of miRNAs sequencing between plasma samples and serum samples in our analysis. Plasma and serum samples were obtained from the same subjects, 105 participants in total. As shown in this figure, for all miRNA reads, the serum sample is significantly higher (A, p-value < 0.05), but for the number of unique miRNAs detected, the plasma is significantly higher than serum samples (B, p-value < 0.05). After preprocessing and normalization procedures including filtering low quality miRNAs and samples, the levels of 241 plasma miRNAs and 210 serum miRNAs were obtained from 76 subjects, among which there are 204 common miRNAs. With significance of p-value < 0.05, the levels of 175 miRNAs were significantly different in two kinds of specimens, of which 30 miRNAs had higher levels in serum than in plasma, while 145 miRNAs had higher levels in plasma than in serum.

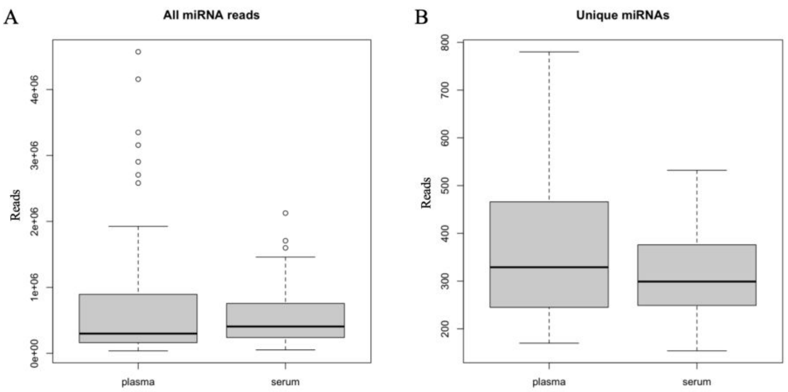

Figure S3. The correlation matrix of miRNAs levels in plasma and serum samples. Pearson coefficients were used to quantify the correlation of the levels of 204 miRNAs in plasma and serum samples from 76 subjects. Each mosaic point represents the correlation coefficient between one miRNA level in 76 plasma samples and one miRNA level in 76 serum samples. From this plot, we can find a fuzzy line in the diagonal, which is the directly comparison between same miRNAs in plasma and in serum. To further confirm the extent of correlation, we used histogram to exhibit the coefficients in the diagonal, please see figure S4.

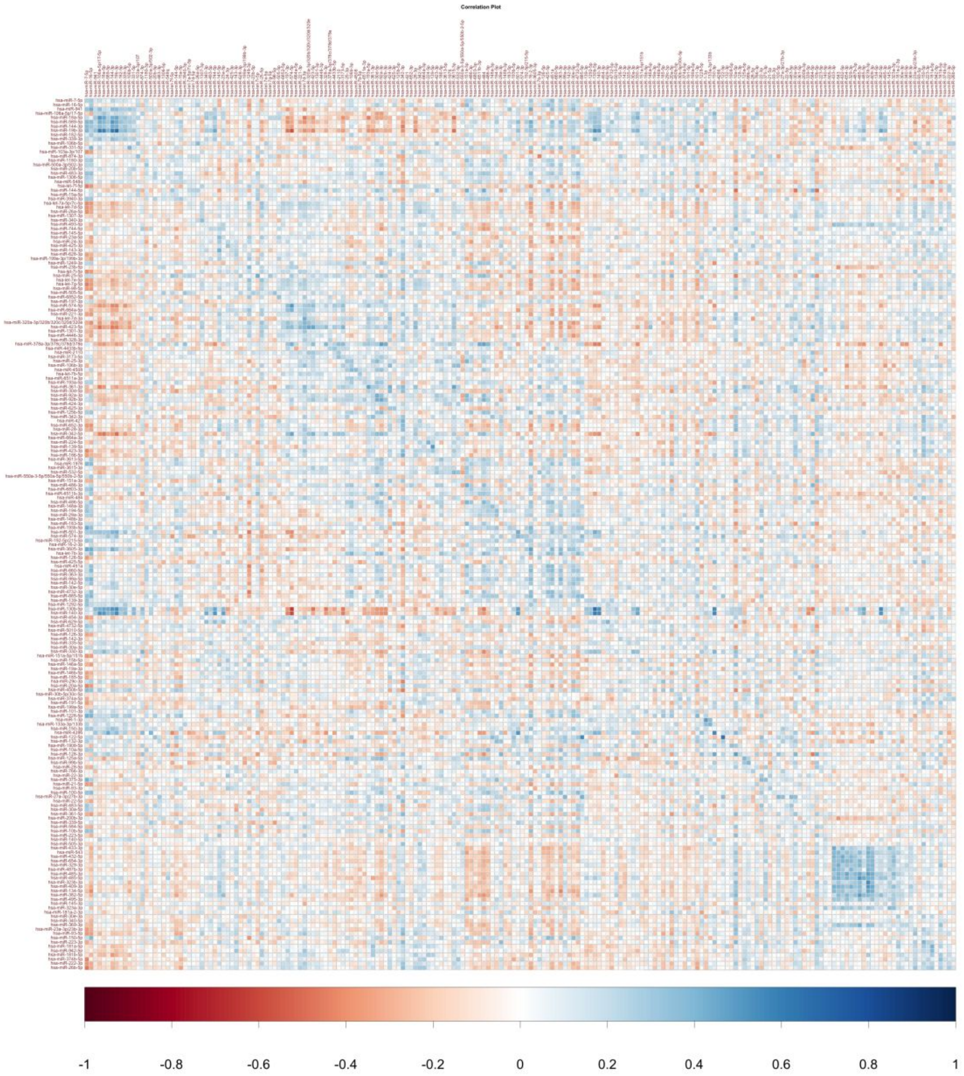

Figure S4. The histogram of correlation coefficients between plasma miRNAs levels and serum miRNAs levels. Only the diagonal of the correlation coefficients matrix was shown (-0.22 < r < 0.68). From this histogram, the coefficients of all miRNAs were lower than 0.8, and only a few miRNAs were higher than 0.5, which suggest a low correlation between plasma miRNAs and serum miRNAs.

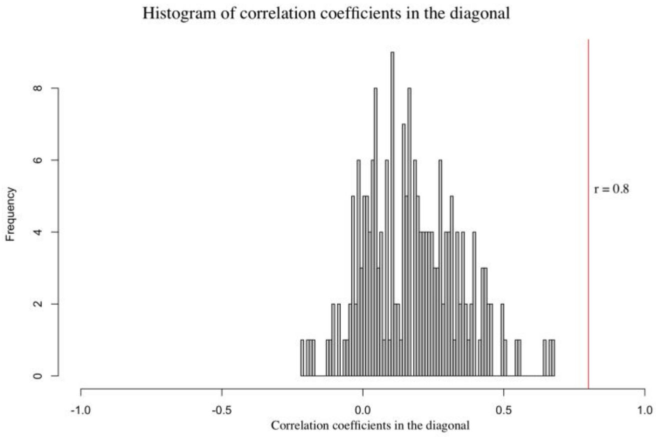

Figure S5. The Area Under the Curve (AUC) scores of cerebrospinal fluid (CSF) biomarkers associated plasma miRNAs in Alzheimer's disease (AD) vs controls (A,B), and in mild cognitive impairment (MCI) vs controls (C,D). From A and C, we found that miR-145-5p has the highest AUC score in both predicting AD and MCI, 0.77 and 0.72 respectively. In B and D, y-axis presents the AUC scores, we randomly selected one miRNA and plot the AUC score, the blue point shows the AUC score of miR-145-5p, both plots show that miR-145-5p has better prediction performance than other miRNAs.

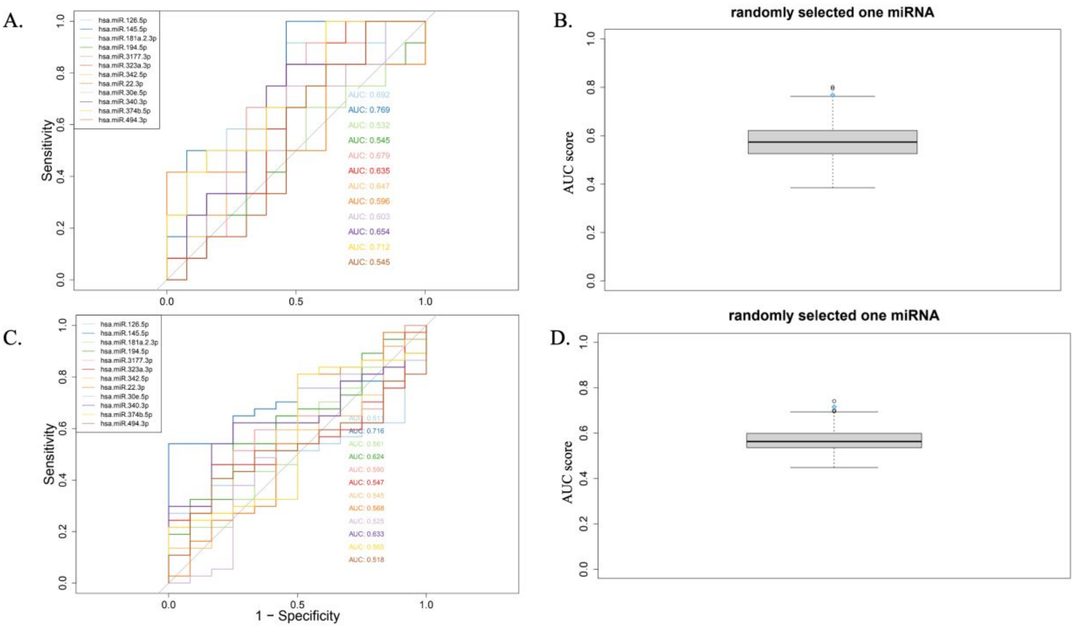

Figure S6. The Area Under the Curve (AUC) scores of different biomarkers in predicting Alzheimer's disease (AD) (A) and predicting mild cognitive impairment (MCI) (B) from controls. In both plots, Aβ represents amyloid β1-42 (Aβ1-42) levels, miR represents miR-145-5p, Magnetic Resonance Imaging (MRI) represents normalized hippocampus volume. In AD samples, miR-145-5p plus Mini-Mental State Examination (MMSE) achieved AUC score of 1.0, which is same with Aβ1-42 plus MMSE, and improved MMSE's performance, which is 0.97 AUC score, and it also improved MRI biomarker (AUC: 0.95). In MCI samples, miR-145-5p plus MMSE had lower AUC score, which is 0.88 AUC score, still

improved MMSE (AUC: 0.85) and the MRI biomarker (AUC: 0.8).

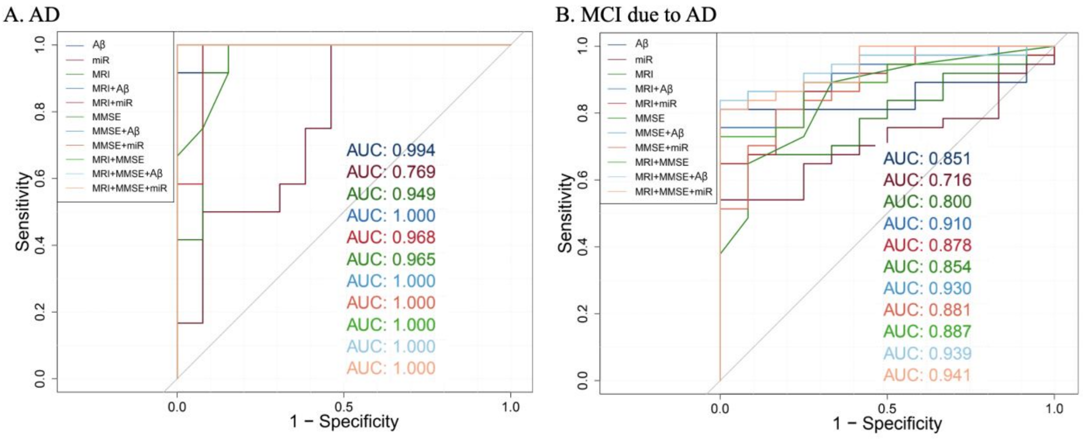

Figure S7. The Area Under the Curve (AUC) scores of different biomarkers in classify Alzheimer's disease (AD) and mild cognitive impairment (MCI). CSF represents cerebrospinal fluid (CSF) biomarker indicates phosphorylated tau181 (P-tau181) levels, miR represents miR-22-3p, Magnetic Resonance Imaging (MRI) represents normalized hippocampus volume. MiR-22-3p alone had better performance than CSF biomarker P-tau181, and this miRNA plus Mini-Mental State Examination (MMSE) achieved AUC score of 0.82, which is better than CSF biomarker plus MMSE, and improved the performance of MMSE and MRI data as biomarker.

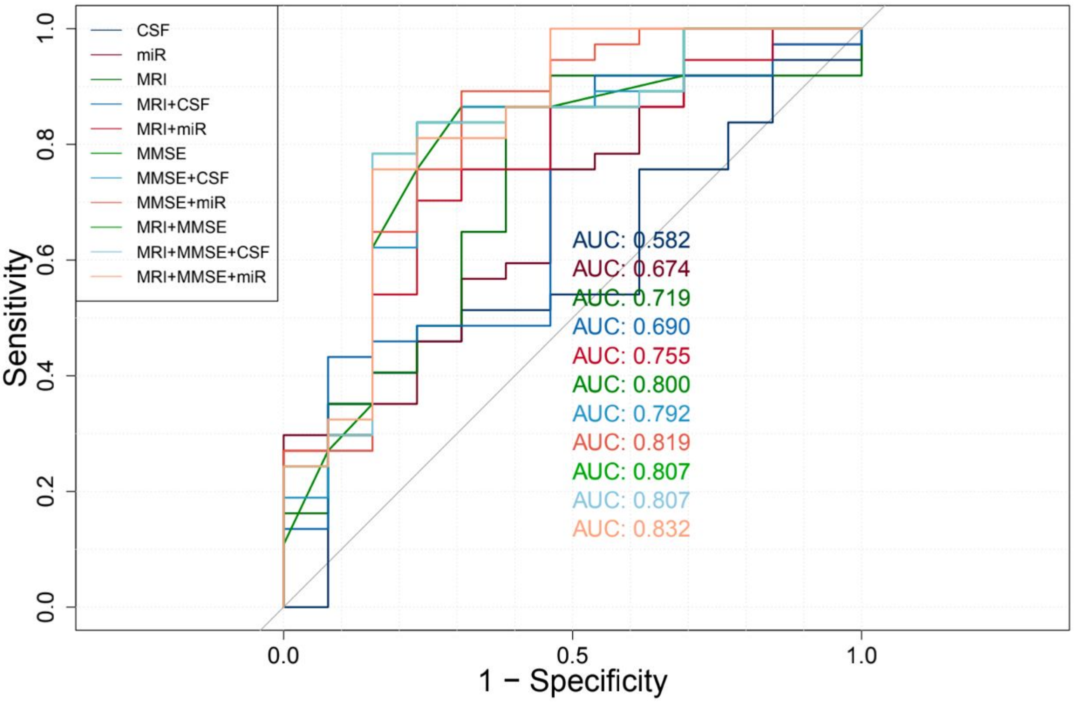

Figure S8. The volcano plots for DEmiRNAs in serum and plasma samples regarding different comparing groups. Three serum differentially expressed miRNAs (DEmiRNAs) (including miR-133a-3p/133b, miR-143-3p, and miR-4433b-5p) were identified in Alzheimer's disease (AD) vs controls, one serum DEmiRNA (miR-885-5p) was identified in mild cognitive impairment (MCI) vs controls (False Discovery Rate (FDR) < 0.05). For these DEmiRNAs, two miRNAs were

identified with Log2FoldChange < -1 (miR-133a-3p/133b and miR-885-5p, marked blue in D and E).

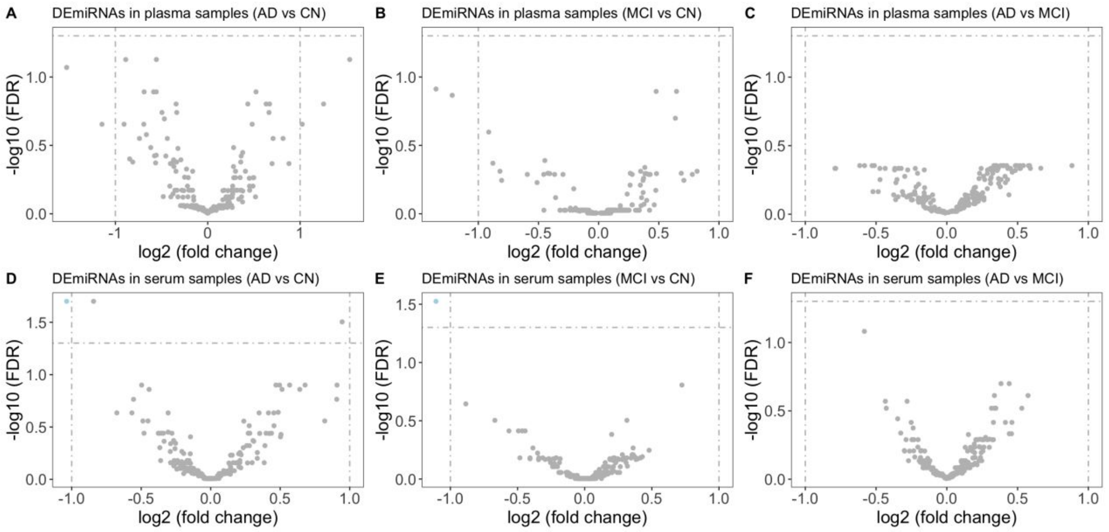

Figure S9. The density plot of miRNA expression data of both plasma and serum samples. A plasma sample, B serum sample. Each line indicates an individual sample.

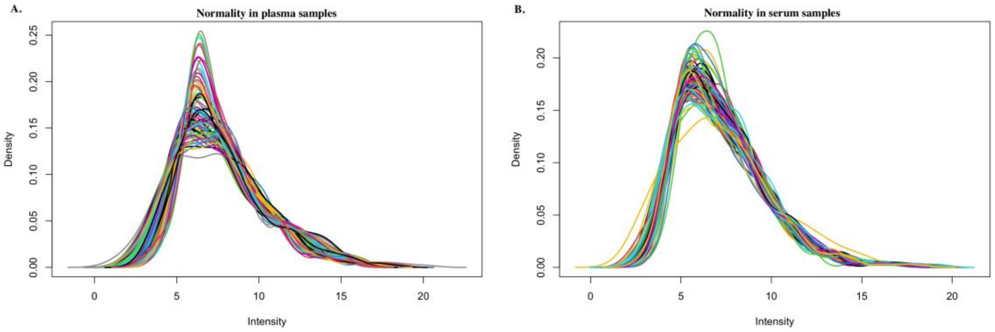

Figure S10. The quantile–quantile (QQ) plots for numeric variables and the volcano plots for categorical variables. In volcano plots, if the p-value (False Discovery Rate (FDR) corrected) is lower than 0.1, it would be marked as blue (decreased levels) or orange (increased levels). A. Plasma samples, therefore the model for plasma is: miRNAs ~ (1 | batch 1) + (1 | batch 2) + Age + Gender + Education + PPI + SNRI + interested variable, among which batch 1 is the year of samples collection, batch 2 is date of RNA isolation and library. B. Serum samples, therefore the model for serum is: miRNAs ~ (1 | batch 1) + (1 | batch 2) + Age + Gender + Education + SNRI + interested variable, among which batch 1 is the year of samples collection, batch 2 is date of RNA isolation and library. PPI is the abbreviation for proton pump inhibitor, SNRI is the abbreviation for serotonin-norepinephrine reuptake inhibitor.

A.

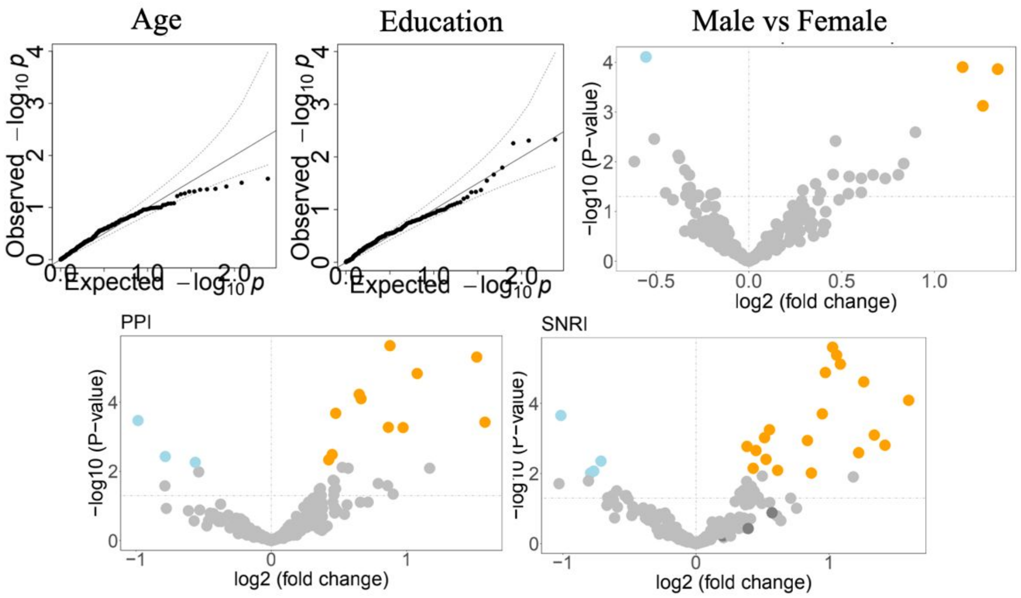
Age
Education
Male vs Female
Observed $-\log_{10} p$
Expected $-\log_{10} p$
-log10 (P-value)
log2 (fold change)
PPI
SNRI

B.

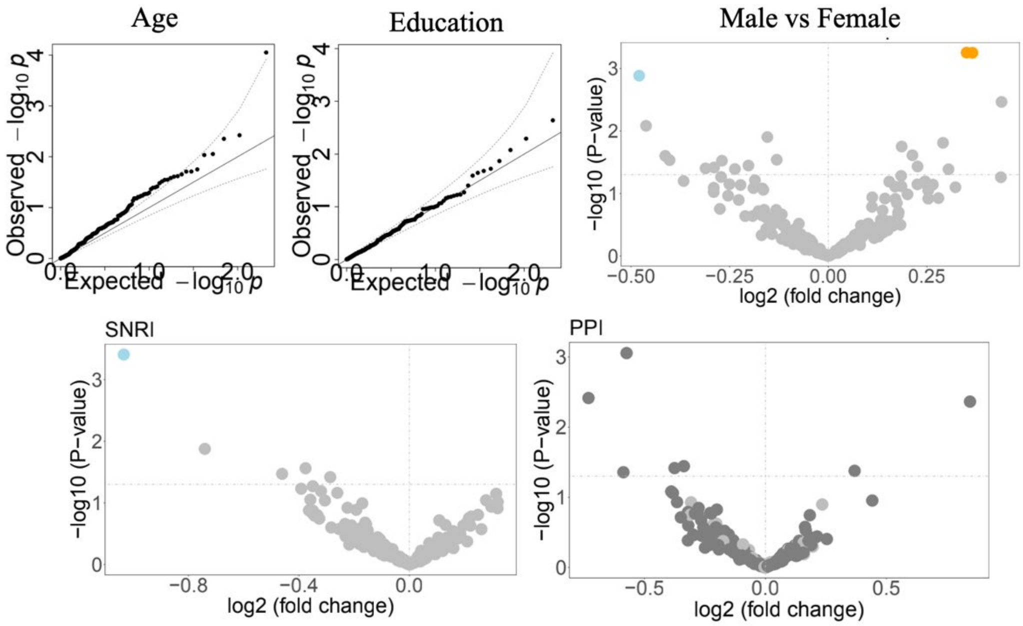
Age
Education
Male vs Female
Observed $-\log_{10} p$
Expected $-\log_{10} p$
-log10 (P-value)
log2 (fold change)
SNRI
PPI
